# A Practical Treatise on the Diseases of the Respiratory Organs; Including Diseases of the Larynx, Trachea, Lungs, and Pleura

**Published:** 1845-02

**Authors:** 


### BIBLIOGRAPHICAL NOTICES.

*A Practical Treatise on the Diseases of the Respiratory Organs; including Diseases of the Larynx, Trachea, Lungs, and Pleura.* By Charles J. B. Williams, M. D., F. R. S., Fellow of the Royal College of Physicians, Professor of the Principles and Practice of Medicine, and of Clinical Medicine, and First Physician to the Hospital University College, London, &c. *With numerous Additions and Notes.* By Meredith Clymer, M. D., Physician to the Philadelphia Hospital, Fellow of the College of Physicians, &c., &c. *With Woodcut Illustrations.* 8vo. pp. 508. Philadelphia: 1845.

Dr. Williams, by position and character, is justly entitled to attention as a pathologist and practised writer. His "Principles of Medicine" we have not held in the high estimation in which it is held by some; yet we never see any thing from him that is not respectable. The basis of the present volume is the Essay on the Diseases of the Organs of Respiration, contributed by Dr. Williams to the "*Library of Practical Medicine,*" edited by Dr. Tweedie. "Written," says the American editor, "several years" [quære, how many?] "subsequently to the 'Lectures on the Diseases of the Chest,' they embody the more mature experience of the author, besides being presented in a more available form." We apprehend that the lapse of time referred to was too short to admit of a much more extended experience. "In issuing them," Dr. Clymer adds, "as a separate publication, various additions became necessary. The whole work has been carefully revised from the Lectures of Professor Williams on these diseases, which have just been completed in the London 'Medical Times.' Much new matter has been added to every section, and several new chapters introduced. In doing this the editor has endeavoured to maintain, as far as possible, the practical and authoritative character of the work; and he has sought to add, not so much that which was new, as that which was valuable, and had received the sanction of the profession."

The essays of Dr. Williams have been for some years before the public, and therefore require no examination from us. The additions of Dr. Clymer appear to be generally judicious. The chief sources from which he has drawn his materials, he remarks, are the works of Louis, Grisolle, Piorry, Rilliet and Barthez, Valleix, &c.; whilst the synoptical tabular statements are modified from those in the Manual on the Physical Diagnosis of the Diseases of the Lungs, of Dr. Walshe.

One or two remarks we may make on the additions of Dr. Clymer. In treating of acute laryngitis, he says, "So soon as it is evident that bloodletting is not telling," [!] "and the moment the skin becomes dusky, tracheotomy should be performed. The operation should be performed, if possible, whilst the patient's strength is yet entire, and before the system is already poisoned by unarterialized blood, and the lungs congested; if it do not save life, it will disarm death of its agony." [?] "Its early performance is urged by Dr. Chapman." p. 124.

And does Dr. Clymer really believe, that the view of Bichat on the "poisonous" effects of unarterialized blood is a just one? This, we thought, had been long abandoned by the wisest and the best; and in regard to the importance of having early recourse to the operation of tracheotomy, we apprehend that Dr. Chapman but adopts the views that have been generally laid down by therapeutists. He does not refer in the most distant degree to his own experience. In his peculiar style—and we cannot commend it—he says, "Twenty-eight well authenticated cases of success have I collected, of which nineteen were in the latter," [laryngitis,] "and eleven in the former affection," [croup.] "No reference now have I to a recent report by the Parisian practitioners of their great success, which, I think, comes to us in too questionable a shape to be trusted." [Why so?] "But I repeat that the operation is not too long to be postponed."

Dr. Clymer evidently—like others—has his more especial favourites, to whom he refers; otherwise why did he not equally allude to authorities for the following pathological suggestions.

"The vocation of the clergy has been thought to render them peculiarly liable to this disease, [chronic laryngitis,] especially in this country, and it has, in consequence, been called the 'clergymen's soar throat.' This peculiar susceptibility from the nature of their pursuits may be doubted. The disease, in fact, to which they, in common with others, seem particularly liable, is a chronic pharyngitis, and is popularly known as bronchitis. On inspection of the pharynx, its lining membrane will be found to be injected, and the follicles greatly enlarged, and resembling split peas." p. 129.

Under aphonia we have the following observations which puzzle us not a little.

"*Hysterical aphonia*, or loss of voice, occurs suddenly, may last for several months or even years, and then disappears as suddenly. A patient in this state, who for some time has been unable to speak above a whisper, may, under the influence of strong moral excitement, recover his natural tone of voice. Recovery may be permanent, or a relapse may take place. This affection is most commonly met with in women, but, according to Sir Benjamin Brodie, it is not unfrequently seen in the male sex, especially in those of the divine profession, probably because they often lead very sedentary lives, and also because in their profession they are called upon to speak in public in a tone raised above the ordinary standard."

Surely Dr. Clymer does not consider that these last are cases of *hysterical aphonia!*

In the treatment of the coryza of infants, remedies are suggested by him which seem to us of more than questionable propriety.

"The infant should not be placed to the breast during a severe catarrh, but should be fed with the spoon on fresh cow's milk, diluted with barley water, or rice water. The vapour of tar often facilitates the secretion from the nostrils in such cases, and may be used by placing a cup half filled with tar in a bowl of boiling water. Hot vinegar or hot water is also useful." p. 185.

Notwithstanding these criticisms, the additions of Dr. Clymer, we repeat, are generally as judicious as they are numerous and useful; and where we differ from him we are not sure that we may not occasionally have misunderstood him. He is, we know, well read; maintains himself *à portée* with the condition of medical science; and, therefore, we are the more surprised to see in him anxiety to cite remarks, which are by no means novel, and are often only reiterated by authors because they may have been employed to depict the existing state of knowledge and received opinions on some subject. We take at random the following exemplification of our remarks, in which Dr. Walshe, (and Dr. Clymer after him,) merely repeats the definition of all modern writers.

"This term," [empyema] "which etymologically (εμπυημα from εν, in, and πυον, pus" [written π̄υον in the work before us,]) "means any internal collection of pus, was restricted by the older writers to purulent accumulation in the chest, a condition which would be more correctly expressed by the term Pyothorax. In practice, however, the word empyema is now generally employed in a wider sense, and is applied to every fluid pleuritic collection, whether purulent, puriform, albumino-serous or otherwise, which manifestly runs a chronic course, and continues stationary, or increases in spite of remedial measures." (Walshe, Cyc. Pract. Surg. P. ix. p. 93.)

The work contains many formulæ written by Dr. Clymer, but not always accurately printed. We wish, that he had adhered to the nomenclature of our Pharmacopœia. It is very desirable, that all writers on this side the Atlantic should adopt one standard, and it is admitted, that no work of the kind is entitled to more consideration than our own.

